# The Concept and the Epidemiology of Diabetic Nephropathy Have Changed in Recent Years

**DOI:** 10.3390/jcm4061207

**Published:** 2015-05-28

**Authors:** Alberto Martínez-Castelao, Juan F. Navarro-González, José Luis Górriz, Fernando de Alvaro

**Affiliations:** 1 Bellvitge´s University Hospital, Carlos III Research Institute, Barcelona 08907, Spain; 2 Candelarias’s University Hospital, Carlos III Research Institute, Tenerife 38010, Spain; E-Mail: jnavgon@gobiernodecanarias.org; 3 Dr. Peset University Hospital, Carlos III Research Institute, Valencia 46017, Spain; E-Mail: jlgorriz@senefro.org; 4 Infanta Sofia Hospital, Carlos III Research Institute, Madrid 28702, Spain; E-Mail: fernandodealvaro2@gmail.com

**Keywords:** diabetes mellitus, epidemiology, diabetic nephropathy, diabetic chronic kidney disease, end-stage renal disease

## Abstract

Diabetes Mellitus (DM) is a growing worldwide epidemic. It was estimated that more than 366 million people would be affected. DM has spread its presence over the world due to lifestyle changes, increasing obesity and ethnicities, among others. Diabetic nephropathy (DN) is one of the most important DM complications. A changing concept has been introduced from the classical DN to diabetic chronic kidney disease (DCKD), taking into account that histological kidney lesions may vary from the nodular or diffuse glomerulosclerosis to tubulointerstitial and/or vascular lesions. Recent data showed how primary and secondary prevention were the key to reduce cardiovascular episodes and improve life expectancy in diabetic patients. A stabilization in the rate of end stage kidney disease has been observed in some countries, probably due to the increased awareness by primary care physicians about the prognostic importance of chronic kidney disease (CKD), better control of blood pressure and glycaemia and the implementation of protocols and clinical practice recommendations about the detection, prevention and treatment of CKD in a coordinated and multidisciplinary management of the DM patient. Early detection of DM and DCKD is crucial to reduce morbidity, mortality and the social and economic impact of DM burden in this population.

## 1. Has the Diabetes Mellitus Epidemiology Changed in Recent Years?

Diabetes Mellitus (DM) is a growing worldwide epidemic. The World Health Report in 1997 projected a continuous growth in the prevalence of DM for the next 20 years [1].

Many studies demonstrate rising prevalence of diabetes worldwide over the past decades. In 2004, Wild *et al*. estimated an increase in the global prevalence of DM from 2.8% in 2000 to 4.4% in 2030 in the world, taking into account the high percentage of undiagnosed diabetes. That means more than 366 million people would be affected by this systemic metabolic disorder [2].

The number of diabetic American adults treated rose more than two fold between 1996 and 2007 (from about 9 million to 19 million). By age groups, the number of diabetic patients increased from 4.3 million among people aged 65 and older; 3.6 million to 8.9 million among adults aged 45 to 64 and 1.2 million to 2.4 million among people aged 18 to 44, as was reported by the Agency for Healthcare Research and Quality [3]. The total cost of DM climbed from $18.5 billion to nearly $41 billion during that time, according to the National Medical Expenditure Panel Survey.

In the year 2008, The Lancet published the global challenge of Diabetes [4]. Danaej *et al*. [5] estimated trends in mean fasting plasma glucose (FPG) and DM prevalence for adults aged 25 years and older in 199 countries and territories. The authors obtained data from health examination surveys and epidemiological studies (370 country-years and 2.7 million participants). For each sex, they used a Bayesian hierarchical model to estimate mean FPG and its uncertainty. In 2008, global age-standardized mean FPG was 5.5 mmol/L for men and 5.42 mmol/L for women, having risen by 0.07 mmol/L and 0.09 mmol/L per decade, respectively. Age-standardized adult diabetes prevalence was 9.8% in men and 9.2% in women in 2008, up from 8.3% and 7.5% in 1980. The number of people with diabetes increased from 153 million in 1980, to 347 million in 2008. They recorded almost no change in mean FPG in East and Southeast Asia and Central and Eastern Europe. Oceania had the largest rise, and the highest mean FPG (6.09 mmol/L, for men; 6.08 mmol/L, for women) and diabetes prevalence (15.5%, for men; and 15.9%, for women) in 2008. Mean FPG and diabetes prevalence in 2008 were also high in South Asia, Latin America and the Caribbean, and central Asia, North Africa, and the Middle East. Mean FPG in 2008 was lowest in sub-Saharan Africa, East and Southeast Asia, and high-income Asia-Pacific. In high-income subregions, Western Europe had the smallest rise, 0.07 mmol/L per decade for men and 0.03 mmol/L per decade for women; North America had the largest rise, 0.18 mmol/L per decade for men and 0.14 mmol/L per decade for women. The conclusions of this study were that glycaemia and diabetes are rising globally, driven both by population growth and ageing and by increasing age-specific prevalence [5].

Abraham *et al*. [6] analyzed trends in diabetes incidence over the previous four decades in USA. The Framingham Heart Study participants, aged 40–55 years and free of diabetes at baseline (*n* = 4795; mean age 45, 3 years; 51.6% women), were followed and screened for diabetes in the 1970s, 1980s, 1990s, and the 2000s. Diabetes was defined as either fasting glucose ≥126 mg/dL or use of oral hypoglycemic agents or insulin. Rates were also calculated among obese, overweight and normal weight individuals. The annualized rates of diabetes per 1.000 individuals were 2.6, 3.8, 4.7, and 3.0 (women) and 3.4, 4.5, 7.4, and 7.3 (men) in the 1970s, 1980s, 1990s, and 2000s, respectively. Compared with the 1970s, the age- and sex-adjusted relative risks of diabetes were 1.37 (95% CI 0.87–2.16; *p* = 0.17), 1.99 (95% CI 1.30–3.03; *p* = 0.001), and 1.81 (95% CI 1.16–2.82; *p* = 0.01) in the 1980s, 1990s, and 2000s, respectively. Compared with the 1990s, the relative risk of diabetes in the 2000s was 0.85 (95% CI 0.61–1.20; *p* = 0.36). The authors concluded that the risk of new-onset diabetes continued to be higher in the 2000s compared with the 1970s. Despite the continuous increase of obesity and adiposity, diabetes incidence stayed unchanged over the past decade.

Regarding Spanish data, Soriguer *et al*. [7] published the Di@bet.es Study in 2011. It was a population-based, cross-sectional, cluster sampling study, targeting the whole Spanish population. Five thousand and seventy-two participants in 100 clusters (health centers or the equivalent in each region) were randomly selected with a probability proportional to population size. Participation rate was 55.8%. Study variables were a clinical and demographic structured survey, lifestyle survey, physical examination (weight, height, BMI, waist and hip circumference, blood pressure) and oral glucose tolerant test (OGTT). Almost 30% of the study population had some carbohydrate disturbance. The overall prevalence of diabetes mellitus adjusted for age and sex was 13.8% (95% CI 12.8, 14.7%), almost half of them had unknown diabetes, 6.0% (95% CI 5.4, 6.7%). The age- and sex-adjusted prevalence rates of isolated impaired fasting glucose (IFG), isolated impaired glucose tolerance (IGT) and combined IFG-IGT were 3.4% (95% CI 2.9, 4.0%), 9.2% (95% CI 8.2, 10.2%) and 2.2% (95% CI 1.7, 2.7%), respectively. The prevalence of diabetes and impaired glucose regulation increased significantly with age (*p* < 0.0001), and was higher in men than women (*p* < 0.001).

In 2012, Polonsky K.S. [8] depicted a spectrum of diabetes quite different from the classical concept of DM. At that time, DM accounted for about 10% of cases, age-adjusted 6.9% of the U.S. population in 2010. Following recent data, DM patients increased from 5.6 million to 20.9 million in the general population. Nearly 27% of people over 65 years of age had DM. If the current trend continues, one in three U.S. adults could have DM in 2050 [9].

In these studies, the vast majority of patients with DM are overweight, having a combination of insulin resistance and impaired insulin secretion.

If we take all these data into account, it seems evident that DM and, specially, type 2 DM, has spread its presence over the world. This may be due to different factors, such as lifestyle changes, increasing obesity and ethnicities, among others.

Whit regard to the impact of immigration on the diabetes incidence and complications, migration flows probably affect diabetes complications. It could vary depending on immigrant’s age at landing, educational level, marital status and neighborhood of settlement. In a recent Canadian study, immigrants with language barriers did not demonstrate higher risk for acute complications, cardiovascular (CV) events or death. However, some factors were related to complications as being elderly, living in a rural region, being unmarried or having a low level of education [10].

An important issue to consider is the change of diabetes-related complications over the last years. The National Interview Health Survey (NHIS) is a North-American multistage study which samples an average of 57.000 adults per year to estimate the health of the U.S. population. Data from the NHIS and the National Hospital Discharge Survey (NHDS) identified five DM-related complications: lower-extremities amputation, acute myocardial infarction, stroke, end-stage renal disease and death from hyperglycaemic crisis. Between 1990 and 2010, the number of adults reporting DM grew from 6.5 million to 20.7 million whereas the U.S. adult population overall increased by approximately 27%. However, the relative risk of events associated to DM went down (amputations from 22.6 to 18.8, end-stage renal disease from 13.7 to 6.1, acute myocardial infarction from 3.8 to 1.8 and stroke from 3.1 to 1.5). Although definitions may differ across countries making comparisons difficult, these findings are consistent with trends in cardio-vascular disease and all-cause mortality reported from 1997 to 2006 in the USA [11].

In 2008, we reported the clinical and social impact of the DM and its complications in Spain, [12]. Mata *et al*. estimated the global cost of DM on €2.132 per patient-year, when micro and macrovascular complications were present [13]. An interesting study of Lorenzo *et al*. in the Canary islands in Spain—an autonomous community with a high rate of ESRD due to diabetes—estimated that reducing the rate of ERC-5 due to diabetes in this community may lead to a decrease of 15 to 25 million € in a three year period [14].

**Figure 1:**
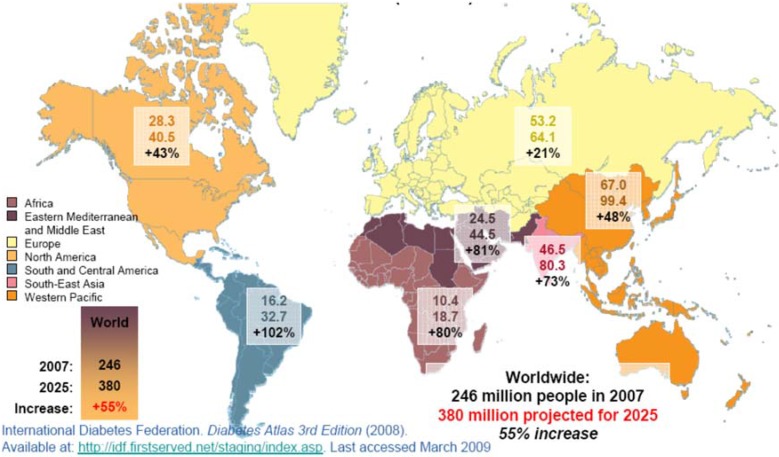
World expansion of diabetes mellitus, International Diabetes Federation [15].

Figure 1 represents the World expansion of diabetes mellitus from 2007 to 2025, according to the International Diabetes Federation [15]. According to the report from The American Diabetes Association (ADA) in 2013, based on the *Economic Costs of Diabetes in the U.S. in 2012* study, the total cost of diagnosed diabetes rose from $174 billion in 2007 to $245 billion in 2012. That study addressed some relevant items such as the increased financial burden, the health resources and the loss of productivity associated with this disease. It also included a detailed breakdown of costs along gender, racial and ethnic lines, and a breakdown of costs on a state-by-state basis. The total estimated cost of diagnosed diabetes in 2012 was $245 billion, including direct medical costs ($176 billion) and lack of productivity ($69 billion) [16].

## 2. A Changing Concept: From Diabetic Nephropathy to Diabetic Kidney Disease (DKD)

Until recent years, diabetic nephropathy was defined by the evidence of a renal disturbance which is characterized by the presence of proteinuria, equal or more than 300 mg/day, in a diabetic patient. Usually, this clinical situation was accompanied by diabetic retinopathy and hypertension, leading to a progressive deterioration in the kidney function. Nevertheless, the absence of diabetic retinopathy does not exclude the diagnosis of DN. The natural history of diabetic renal disease differs between type 1 and type 2 DM. The five classical stages described in type 1 DM [17,18], may not occur in type 2 DM [19,20], because sometimes type-2 DM is diagnosed after other connected disorders as hypertension, proteinuria or renal failure.

The development of microalbuminuria and the progression to overt proteinuria are the most common clinical features. However, in contrast to the predictions of the classical model for kidney disease involvement, a considerable percentage of patients with diabetes and impaired renal filtration do not have substantially elevated urinary protein excretion rates. All studies on this subject are observational and most lack biopsy data. A well-designed biopsy study and a series of intervention trials are needed to fully understand this entity [21].

With regard to this aspect, Tervaert *et al*. reported in 2010 a new pathology classification of the diabetic kidney lesions where the authors insisted on the existence of some forms of kidney damage with primary involvement of tubules, interstitium and/or the vessels, far away from the classical nodular or global glomerulosclerosis [22] (see Table 1).

All these new findings lead to a change in diabetic nephropathy concept, shifting from the classical one “diabetic nephropathy” to the new one “diabetic chronic kidney disease” (DCKD).

## 3. End-stage Renal Disease in Diabetic Patients: Has the Presence of DCKD Changed in Parallel to the DM Development?

Recent data showed how primary and secondary prevention were the key to reduce CV episodes and improve life expectancy in diabetic patients [23]. Does the decrease in this competing risk lead to an increase in the rate of end stage kidney disease? The answer is probably no.

In 2005, we estimated around 33,000 type 1 and 405,000 type 2 diabetic patients with some form of DN, from microalbuminuria to ERSD in Spain [24].

In 2013, our group also published the results from Catalonia, Spain. They showed a decrease from 6456 per million population (p.m.p.) in 2002 to 600 p.m.p. in 2010 in ESRD (7% reduction of adjusted rate) [25]. Data from the Spanish Society of Nephrology (S.E.N.), recorded in the annual renal registry, also showed a stabilization of DM as a cause of CKD requiring renal replacement therapy (RRT) in the last four years (incidence: 24.97% in 2011, 24.91% in 2012, 24.90% in 2012 and 24.71% in 2013 and rate p.m.p: 31.6 in 2010, 32.1 in 2011, 32.9 in 2012 and 31.9 in 2013). Although DM continuous to be the first cause of ESRD in Spain—as in the whole world—we have observed a clear stabilization of ESRD due to DM compared to other causes of CKD-5 [26].

**Table 1:** Pathologic classification of diabetic nephropathy (modified from Tervaert *el al.* [22]).

Classes of Glomerular Lesions	Description
Class I	Glomerular basement membrane thickening
Class II	Mesangial expansion, mild (IIa) or severe (IIb)
Class III	Nodular sclerosis (Kimmelstiel-Wilson lesions)
Class IV	Advanced diabetic glomerulosclerosis
**Tubulointerstitial Lesions**	**Scores**
IFTA	
No	0
<25%	1
25%–50%	2
>50%	3
Interstitial inflammation	
No	0
Related to IFTA	1
Areas without IFTA	2
**Vascular Lesions**	
Arteriolar hyalinosis	
No	0
1 area	1
>1 area	2
Presence of large vessels	
Arteriosclerosis	
No	0
Intimal thickening less than thickness of media	1
Intimal thickening greater than thickness of media	2
Nondiabetic Glomerular Lesions	

Such findings were confirmed in recent studies. The ESRD report in U.S.A. showed a stabilization of the percentage of ESRD in almost 190 p.m.p., from 2002 to 2003 [27]. Besides, Burrows *et al*. reported 2.9% annual decrease in overall incidence of ESRD due to DM, from 1996 to 2006 [28]. When considering the data from Australia and New Zealand Dialysis and Transplant (ANZDATA) Registry Grace *et al*. also described the stabilization in age-specific incidence rates in most groups during the past 5 years [29].

Many factors may explain this paradox, a decrease in ESRD secondary to diabetic nephropathy and an increase in the rates of DM in the general population concurrently. An earlier diagnosis and better management of this pathology based on a multidisciplinary approach of the different professionals involved may be an explanation. The earlier and better control of CKD progression risk factors as well as the widespread utilization of renin-angiotensin-aldosterone blockers (RAAB) play a decisive role.

In addition to that, other factors have probably influenced the decline in the incidence in both diabetic nephropathy and ESRD of the other etiologies. These factors include the increased awareness by primary care physicians about the prognostic importance of CKD and its consequences. It is noteworthy that a better control of risk factors for DCKD progression has been achieved by primary care doctors in the last decade, especially regarding blood pressure [30] and glycaemic control [31]. Finally, the scientific societies have played an important role in the dissemination of protocols and clinical practice recommendations about the detection, prevention and treatment of chronic kidney disease and DCKD which undoubtedly have influenced the stabilization of the incidence [32,33].

Even so, and in agreement with Couchoud and Villar [34], epidemiological studies must be interpreted cautiously and bias should be identified: renal biopsies are performed only in a small percentage of patients (10%–20%) [35]; diabetic nephropathy and hypertensive changes are likely to coexist, as the Tervaert classification has shown; variations among nephrologists reporting DM as a primary cause of ESR go from a conservative approach to a more simplistic interpretation; and great variations may exist among countries. For example, DM was reported as a primary renal disease in only 56% of incident ESRD patients with associated type 2 DM in the French Renal Epidemiology and Information Network (REIN) Registry in 2010 [30], but also in 74.1% of such patients in the ANZDATA Registry in 1991–2005 [30]. It seems obvious that a trend towards a more specific approach in coding the underlying nephropathy could also explain some differences in the DM-DCRD paradox.

The stratification of new incident patients according to demographic variations is another interesting point to take into account. Increasing age, population size and residual disease-related effect are factors connected to ESRD incidence in DM [29].

The results analyzed in our exposition need to be confirmed and further prospective studies in populations would be useful to evaluate combined strategies.

## 4. Conclusions and Implications

Despite the growing DM population, a slowdown in DKD progression seems to be evident. Early detection of both DM and DKD are crucial to reduce complications, morbidity and mortality as well as the social and economic impact of DM burden in this population. It is also crucial to improve the survival of these patients once they are under RRT.

There is still a long way towards improvement, but the observed trend probably reflects that an earlier application of preventive measures and treatment and, of course, a coordinated and multidisciplinary management of the patient with DM, with an earlier implementation of guidelines and clinical recommendations, are the key for the equity in the access to the therapeutic options that may have a positive impact on patients’ outcomes.
